# Uncertain Emotion Discrimination Differences Between Musicians and Non-musicians Is Determined by Fine Structure Association: Hilbert Transform Psychophysics

**DOI:** 10.3389/fnins.2019.00902

**Published:** 2019-09-18

**Authors:** Francis A. M. Manno, Raul R. Cruces, Condon Lau, Fernando A. Barrios

**Affiliations:** ^1^School of Biomedical Engineering, Faculty of Engineering, University of Sydney, Sydney, NSW, Australia; ^2^Department of Physics, City University of Hong Kong, Hong Kong, China; ^3^Instituto de Neurobiología, Universidad Nacional Autónoma de México, Querétaro, Mexico

**Keywords:** emotion, psychophysics, modulation, fine structure, envelope, frequency, amplitude

## Abstract

Humans perceive musical sound as a complex phenomenon, which is known to induce an emotional response. The cues used to perceive emotion in music have not been unequivocally elucidated. Here, we sought to identify the attributes of sound that confer an emotion to music and determine if professional musicians have different musical emotion perception than non-musicians. The objective was to determine which sound cues are used to resolve emotional signals. Happy or sad classical music excerpts modified in fine structure or envelope conveying different degrees of emotional certainty were presented. Certainty was determined by identification of the emotional characteristic presented during a forced-choice discrimination task. Participants were categorized as good or poor performers (*n* = 32, age 21.16 ± 2.59 SD) and in a separate group as musicians in the first or last year of music education at a conservatory (*n* = 32, age 21.97 ± 2.42). We found that temporal fine structure information is essential for correct emotional identification. Non-musicians used less fine structure information to discriminate emotion in music compared with musicians. The present psychophysical experiments revealed what cues are used to resolve emotional signals and how they differ between non-musicians and musically educated individuals.

## Introduction

The process of resolving emotions has been described as the optimization of an economic choice (Seymour and Dolan, 2008). Experimentally, emotions are selected due to their perceived certainty and robust differentiation (Russell, 1980; Barrett, 1998; LeDoux, 2000; Phan et al., 2002; Koelsch, 2014). Few studies have analyzed uncertain emotions or the psychoacoustic cues that endow sound with emotion. Here, we sought to identify the sound attributes involved in musical emotion discrimination and to determine if non-musicians and musicians perceive emotional sound cues differently. We were interested in emotional uncertainty, where the perception of sound attributes is difficult to distinguish.

### Cues in Emotion

Music is transmitted through temporal fine structure (TFS) and envelope (ENV) modulations, which are the perceived changes of the sound in amplitude and frequency, respectively. However, the exact contributions of TFS and ENV used to resolve emotion in music are unknown. Although the influence of pitch on emotional content of speech is well known (Lieberman and Michaels, 1962; Wildgruber et al., 2005), its contribution to music is less clear (Scherer, 1995; Coutinho and Dibben, 2013). Most studies have concentrated on emotion in speech (e.g., since very early on: Fairbanks and Pronovost, 1938; Fairbanks, 1940; Lieberman et al., 1964) or the cues (TFS or ENV) that confer intelligibility to speech (Licklider, 1946; Lieberman and Michaels, 1962; Williams and Stevens, 1972; Frick, 1985; Drullman et al., 1994, 1996; Drullman, 1995; Shannon et al., 1995; Leinonen et al., 1997). Using a Hilbert transform, which allows the signal to be deconstructed and reconstructed into its individual frequency and amplitude time components (King, 2009), researchers found that ENV was most important for speech reception, whereas TFS was most important for pitch perception (Smith et al., 2002). Several follow-up studies have corroborated the importance of ENV for speech intelligibility, in addition to the importance of TFS features (Zeng et al., 2004, 2005; Davidson et al., 2009; Fogerty, 2011; Swaminathan and Heinz, 2012; Apoux et al., 2013; Shamma and Lorenzi, 2013; Moon et al., 2014; Swaminathan et al., 2014). Unfortunately, the sound properties (ENV and TFS) that confer emotion to music have been less studied than the sound properties conveying emotion in speech, i.e., prosody (Hodges, 2010; Coutinho and Dibben, 2013). Therefore, a primary aim for the present experiment was to discern which and how the attributes in sound endow music with emotion.

### Differing Ability to Discriminate Emotions in Auditory Cues: Emotional Resolvability Differences

Musicians have an enhanced auditory perception for several acoustic features, such as the ability to learn lexical tones (Wong et al., 2007), enhanced audiovisual processing (Musacchia et al., 2007), better speech-in-noise perception (Bas̨kent and Gaudrain, 2016; Coffey et al., 2017), better pitch discrimination thresholds (Micheyl et al., 2006), and superior frequency discrimination (Liang et al., 2016; Mandikal Vasuki et al., 2016; Madsen et al., 2017) compared with non-musicians. Musical training and experience shape linguistic patterns (Wong et al., 2007) and enhance speech-in-noise discrimination (Parbery-Clark et al., 2009b), altering brainstem and cortical responses to musical and non-musical acoustic features (Parbery-Clark et al., 2009a; Kraus and Chandrasekaran, 2010; Strait et al., 2010). Musicians possess different auditory perceptual abilities than non-musicians; hence, a musician’s ability to discriminate emotion in sound when linked to TFS or ENV changes should also differ from their non-musician colleagues.

### Speech and Music Relations

There are several dimensions, models, and psychoacoustic features which are used to categorize emotion in music (Russell, 1980; Schubert, 2004, 2013; Gabrielsson and Lindstrom, 2010; Eerola and Vuoskoski, 2011; Eerola, 2012). Psychophysical studies (i.e., frequency and amplitude features) of emotion make up less of the literature than other aspects (i.e., anxiety, arousal, etc., Hodges, 2010). For example, Gingras et al. (2014) found that intensity and arousal ratings in music-induced emotion were largely unaffected by amplitude normalization, suggesting that additional acoustic features besides intensity could account for the variance in subjective arousal ratings. Further, some of the same neurobiological mechanisms underlying emotion in music also subserve the emotion in speech (Fitch, 2006; Juslin and Västfjäll, 2008; Kotz et al., 2018). This is important as several studies give clues as to how uncertain emotion in music might be perceived. For example, Shannon et al. (1995) found speech recognition primarily utilized temporal cues with a few spectral channels. Further to that stated above, Smith et al. (2002) found ENV is most important for speech reception, and TFS most important for pitch perception. Several follow-up studies have corroborated the importance of ENV for speech intelligibility up to a certain number of bands including aspects of TFS (Zeng et al., 2004, 2005; Davidson et al., 2009; Fogerty, 2011; Swaminathan and Heinz, 2012; Apoux et al., 2013; Shamma and Lorenzi, 2013; Moon et al., 2014; Swaminathan et al., 2014). Although the emotion carried in speech is a similar, but different auditory perception than music, resolving emotion in music will aide our understanding of identifying emotions in sound.

### Present Experimental Aims

Our aim was to conduct a psychoacoustic experiment to investigate certain and uncertain emotion in musical sound. We sought to determine which cues are used to resolve emotional signals (Pfeifer, 1988). Moreover, we studied the differences between musicians and non-musicians. To accomplish these aims, we decomposed happy and sad musical stimuli in TFS or ENV using a Hilbert transform (Smith et al., 2002). The process yielded stimuli with increasing decomposition in TFS and ENV, and then we explored the different degrees of emotional certainty they conveyed. Certainty was defined as the ability to identify the decomposed stimuli based on their unaltered forms. Happy and sad stimuli with varying degrees of decomposition were presented in a forced-choice discrimination task. *First*, we expected that decomposing TFS or ENV information essential to determining emotionality in sound would reveal which cue was more important for emotion discrimination. *Second*, we expected that segregating participants by their identification with the original excerpt into good and poor performers, based on the reported classification (Peretz et al., 1998, 2001; Gosselin et al., 2007; Khalfa et al., 2008; Hopyan et al., 2016), would result in different emotional resolvability curves. *Third*, we expected that assessing musicians in their first year of study in the conservatory compared to those in their last year would reveal differences in emotional resolvability based on their musical education. *Lastly*, comparing non-musicians to musicians would reveal differences in emotional resolvability based on musical experience. Our main aim was to understand the cues used to resolve emotional signals.

## Materials and Methods

The experiment included both non-musicians and musicians who were studying music at a conservatory as participants. Participants with musical experience were from a musical conservatory located in Querétaro, Mexico. All data, sound files, and scripts are available at www.fmanno.com, the Open Science Framework (Manno et al., 2018, 2019) and GitHub^1^. Supplementary Data Sheet S1 contains extended analyses and Supplementary Data Sheet S2 contains the original data. The research protocol was approved by the Internal Review Board of the Instituto de Neurobiología, Universidad Nacional Autónoma de México in accordance with the Declaration of Helsinki, 2013. Informed consent (verbal and written) prior to undertaking the experiment was granted and abided by as set forth in the Ethical Principles of the Acoustical Society of America for Research Involving Human and Non-human Animals in Research and Publishing and Presentations.

### Study Participants

Participants were recruited from a local university (Natural Sciences and Musical Conservatory), and final participants were randomly selected from approximately 320 individuals (with the sample not differing from the population group). All individuals were native Spanish speakers. The present study included 64 individuals divided equally into 8 groups (Table 1). Non-musician participants were selected and classified as poor and good (*n* = 32, age 21.17 ± 2.63 SD), based on their performance in the Montreal Emotional Identification Task using greatest mean spread between the groups as the separation metric (Peretz et al., 1998, 2001; Gosselin et al., 2007, 2011; Khalfa et al., 2008; Hopyan et al., 2016). Poor and good participants did not have musical training or education, nor did this cohort play instruments. Poor and good participants were a separate sample from musicians. Musicians were separated based on musical education as first-year (low education) and last-year (high education) students at the conservatory (*n* = 32, age 21.97 ± 2.42). Musically educated participants were recruited from the wind and string sections of the conservatory. All volunteers were free of contraindications for psychoacoustic testing. Prior to undertaking the emotional resolvability experiment, participants had audiometric testing to confirm hearing within normal limits.

**TABLE 1:** Participant data.

	**Non-musicians**				**Musicians**			
	**Good performer**		**Poor performer**		**First year music**		**Last year music**	
**Sex**	**Male**	**Female**	**Male**	**Female**	**Male**	**Female**	**Male**	**Female**
Number	*n* = 8	*n* = 8	*n* = 8	*n* = 8	*n* = 8	*n* = 8	*n* = 8	*n* = 8
Age (years)	20.16 ± 2.11	22.64 ± 3.58	21.11 ± 2.27	20.74 ± 2.11	20.89 ± 1.74	20.56 ± 1.17	23.16 ± 2.01	23.27 ± 3.34
Range (years)	18–25	9–29	19–26	19–25	19–24	19–23	20–26	20–30
Music education	None	None	None	None	2 ± 0	2 ± 0	8.38 ± 2.26	7.12 ± 2.1
Correct identification	97.66 ± 2.21%	95.31 ± 2.36%	80.08 ± 2.86%	80.86 ± 4.24%	93.36 ± 2.00%	92.58 ± 5.52	89.45 ± 3.31%	91.02 ± 4.24%
Total (*n* = 32)	31.25 ± 0.66	30.50 ± 0.71	25.62 ± 0.86	25.88 ± 1.27	29.88 ± 0.64	29.62 ± 1.65	28.62 ± 0.99	29.12 ± 1.27

*Group (good, poor, first year music education or last year music education) by sex (male and female). Years of music education ± SD years. Correct identification – indicating the percent identification based on the original classification of the Montreal Emotional Identification Task. Total – indicating the total number of stimuli presented with the column representing the number out of the total.*

*Audiometric testing* consisted of the examiner presenting to the participants a series of pure tones from 400 to 8,000 Hz, in addition to linear sweeps, log sweeps, and white noise in the same frequency range. The sound pressure level (SPL) decibels (dB) was modulated from 20 to 60 SPL dB. The participants included in the study confirmed hearing the series of audiometric presentations. Participants who did not confirm hearing these tones were excluded.

### Acoustic Stimuli

**FIGURE 1:**
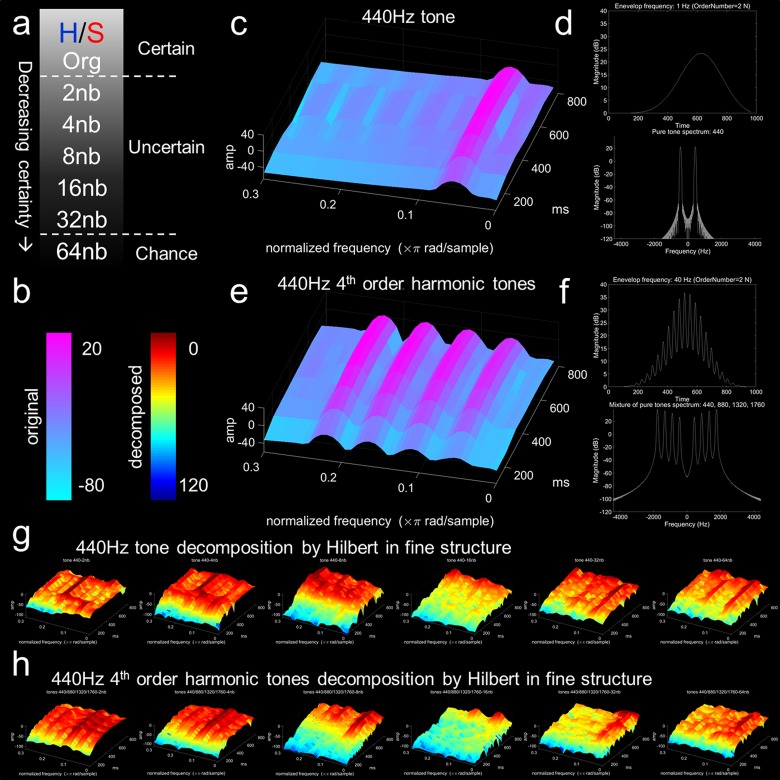
Experimental design and example of the sound decomposition. **(a)** Happy (H)/sad (S) stimuli with decreasing emotional certainty by decomposition. Original excerpts were taken from the Montreal Emotional Identification Task (Peretz et al., 1998; Peretz et al., 2001; Gosselin et al., 2007; Khalfa et al., 2008; Hopyan et al., 2016). Decompositions are based on perceptible categorization as happy or sad, which pertain to certain (org – original) and increasingly uncertain (2nb, 4nb, 8nb, 16nb, 32nb, 64nb decompositions). **(b)** Color bar for coding spectrogram in original (cool color bar) and decomposed (jet color bar) stimuli (simple stimuli example decomposition). Color bar represents normalized power/frequency (dB/Hz) or amplitude (sound pressure level dB) by fine structure components (Hz). All spectrogram plots contain magnitude (dB) on *z*-axis, normalized frequency (×π rad/sample) on *x*-axis, and time in milliseconds (ms) on *y*-axis. **(c)** Single 440 Hz tone. **(d)** Upper panel, single tone with extracted envelope at 1 Hz (order 2N Butterworth filter) with magnitude on the *y*-axis and time on the *x*-axis. Lower panel, fast Fourier transform plot of single tone 440 Hz with magnitude on *y*-axis and frequency (hertz, cycles per second) on *x*-axis. **(e)** Complex 440 Hz 4th order harmonics with 440, 880, 1320, and 1760 Hz components. **(f)** Upper panel, complex tone with extracted envelope at 40 Hz (order 2N Butterworth filter) with magnitude on the *y*-axis and time on the *x*-axis. Lower panel, fast Fourier transform plot of complex 440 Hz 4th order harmonic tones with magnitude (dB) on *y*-axis and frequency hertz on *x*-axis. **(g)** Hilbert decomposition of single 440 Hz tone fine structure with complex 4th order harmonics envelope. **(h)** Hilbert decomposition of complex 4th order harmonics fine structure with single 440 Hz tone envelope. Progression from left to right for both panels **(g,h)** represent the Hilbert transformation for this simple example with 2nb, 4nb, 8nb, 16nb, 32nb, and 64nb decompositions. The simple example demonstrates how a complex acoustic stimulus that is categorized as emotional can be decomposed.

The 32 original acoustic stimuli classified as sad or happy were taken from a previous study [Montreal Emotional Identification Task^2^; Supplementary Table S1 (Peretz et al., 1998, 2001; Gosselin et al., 2007, 2011; Khalfa et al., 2008; Hopyan et al., 2016)]. Half of the stimuli in the repertoire evoked a sense of happiness (major mode with a median tempo of 138 beats per min, bpm), and the other half evoked a sense of sadness (minor mode with a median tempo of 53 bpm) (Peretz et al., 2001). The altered excerpts had the same frequency and amplitude values in terms of pitch and duration as was found in the original stimulus. The sound files of classical music were processed in MATLAB to curtail length to 3-s (in order to present the entire battery of stimuli within an hour period), restricted in frequency/amplitude range for presentation (to reduce noise in the Musical Instrument Digital Interface (MIDI) file), and analyzed spectrally for differences in TFS or ENV (see Figure 1). Original and altered stimuli were presented with MATLAB (Statistics Toolbox Release 2012b, The MathWorks Inc., Natick, MA) using the Psychophysics Toolbox extension^3^ (Manno et al., 2018, 2019). Sound level was adjusted before psychoacoustic testing as described above.

### Acoustic Stimuli Decomposition

Happy and sad stimuli were decomposed by a Hilbert transform in order to derive the altered excerpts (Smith et al., 2002; Moon et al., 2014). The decomposition process associated the acoustic aspects of emotion (happy or sad) with TFS or ENV. The ENV is represented as the magnitude of the Hilbert transform and TFS is the phase (Smith et al., 2002; King, 2009; Moon et al., 2014). Here, we created band-decomposed hybrid stimuli as mixtures of the happy and sad sounds by equal bandwidth steps. Cutting frequencies to 80, 260, 600, 1240, 2420, 4650, and 8820 Hz created six bands of decomposition: 2nb, 4nb, 8nb, 16nb, 32nb, and 64nb. Here, “nb” means number band decomposition as in the original description (Smith et al., 2002). An increase in band decomposition results in decreasing emotional resolvability for the original stimuli (Smith et al., 2002). The entire set of 224 decomposed stimuli and a script demonstrating the Hilbert transform process (Hilbert Explanation) can be found at https://osf.io/8ws7a (Manno et al., 2019). In our case, the Hilbert decomposition resulted in hybrid acoustic sounds and emotional resolvability was effectively tied to TFS or ENV in equally spaced decreasing bandwidths (Smith et al., 2002; Moon et al., 2014). For signal decomposition in the present study, the Hilbert transform *y*(*t*) in the time domain is related to real function *x*(*t*) by the analytic signal *A*(*t*) = *x*(*t*) + i*y*(*t*), with i = (–1)^1/2^. The Hilbert ENV is the magnitude of the analytic signal *|A*(*t*)*|* = ((*s*_*r*_(*t*))^2^ + (*s*_*i*_(*t*))^2)1^*^/^*^2^ and the Hilbert TFS is cos φ(*t*), where φ(*t*) = arctan(*s*_*r*_(*t*)*/s_*i*_*(*t*)) is the phase of the analytic signal (Smith et al., 2002; King, 2009). If the real (r) part pertains to cosine of the frequency contained within the signal and the imaginary (i) part pertains to sine of the frequency contained within the signal, the magnitude of the amplitude is related by the value of the cosine and sine of the signal (King, 2009). The decomposition process has been previously elaborated on for various signal-processing purposes (Oswald, 1956; Smith et al., 2002; Binder et al., 2004; King, 2009; Moon et al., 2014).

### Hybrid Stimuli Example

Recombined hybrid stimuli with differing combinations of ENV and TFS from either emotion category were presented in a happy/sad descending two-interval forced-choice discrimination task (Figures 1a,b). For visualization of the Hilbert process, we created two stimuli, one pure tone (440 Hz, Figure 1c), and a harmonic series of the pure tone (440, 880, 1320, and 1760 Hz, Figure 1e). Both stimuli underwent TFS and ENV extraction by the Hilbert transformation and decomposed into hybrid stimuli for each band of decomposition. Figure 1g presents the Hilbert transformation of the pure tone in TFS combined with the harmonic series stimuli in ENV. Figure 1h presents the Hilbert transformation of the harmonic series stimuli in TFS combined with the pure tone in ENV. From left to right for Figures 1g,h, band decomposition increases from 2nb to 64nb. The pure tone and the harmonics series were given an amplitude double their predecessor (t), starting with 10 dB SPL (decibels sound pressure level), a phase (π/2)/*t* change from its lower harmonic, and separate durations. For the representation, spectrogram plots contained magnitude (dB) on the *z*-axis, normalized frequency (×π rad/sample) on the *x*-axis, and time in milliseconds (ms) on the *y*-axis. Spectrograms and acoustic stimuli were normalized across power/frequency (dB/Hz) and amplitude (dB SPL) by fine structure components (Hz).

### Happy/Sad Forced-Choice Discrimination Task

A forced-choice discrimination task was conducted where participants were required to respond to the acoustic stimuli indicating if they perceived them as happy or sad. The entire repertoire of original and band-decomposed hybrid stimuli was utilized for the present experiments (see Supplementary Music Files at https://osf.io/8ws7a). The participants were presented with all the stimuli and asked to classify them as happy or sad (Smith et al., 2002: Moon et al., 2014). If an original sound was happy, decompositions were categorized as happy and the identification was deemed correct for happy and incorrect for sad. Participants were handed a sheet consisting of 32 rows and 7 columns (see Supplementary Music Matrix at https://osf.io/8ws7a). The columns in the Supplementary Music Matrix are organized as original stimuli presentation, followed from left to right by stimuli decompositions (2nb, 4nb, etc.). The trial was organized by random presentation followed by increasing decompositions. Decompositions in TFS were presented starting with their original unaltered form and continuing through 2nb to 64nb decompositions (Figure 1a).

### Statistical Analysis

#### Average Discrimination Curves by TFS

Average response calculations were derived for TFS for happy and sad musical stimuli. The average discrimination curves were percent identification of the response based on original categorization (found in TFS for all original stimuli). Curves were analyzed independently for non-musician poor and good performers separated by male and female, in addition to first-year (low education) and last-year (high education) musicians separated by male and female.

#### Group Differences

Discrimination curves between groups were tested for significance with an ANOVA and follow-up *t*-test. The discrimination normalized ratio for identification of the stimuli as happy or sad was calculated by determining the percent identification of happy or sad over its opposite stimuli discrimination. Measures of discriminability (D’), and the corrected non-parametric measure of discriminability (A’), were utilized for determining differences in emotional resolvability (Stanislaw and Todorov, 1999; Verde et al., 2006). These measures provide an estimation of signal from noise and determination of the threshold response for the acoustic emotional stimuli (Green, 1960; Swets, 1961, 1986; Swets and Sewall, 1961; Swets et al., 1961). The interest in determining a threshold response for an acoustic emotional stimulus is to ascertain when an emotional stimulus becomes non-emotional (i.e., threshold). Here, we group averaged identifications; however, individual values were calculated by stimuli.

#### Normalized Benefit of TFS and ENV

The difference between TFS or ENV emotion discrimination was examined as the ratio between the perception of one emotion (using TFS or ENV) versus the perception of the other emotion (using TFS or ENV). The ratio was calculated by the normalized benefit of TFS or ENV to the emotion discrimination curve (originally calculated for the visual contribution to speech in noise; Sumby and Pollack, 1954; Meister et al., 2016). The adopted formula was: TFS benefit = (TFS – ENV)/(1 – ENV) or ENV benefit = (ENV – TFS)/(1 – TFS), to compare both the TFS and ENV contributions, respectively, on the scale of +1 to –1. If the percent difference, normalized by each contribution result, was a positive value, this represented benefit to perception. Conversely, a negative value indicated lack of contribution to perception.

#### Discriminant Analysis

A canonical discriminant analysis was used to determine the weight of our variables (nb0, nb2, nb4, etc.), which best separated our different groups (poor performer, good performer, first year musician, last year musician). The two generalized canonical discriminant analyses (one for happy and one for sad) were computed using the multivariate linear model Group ∼ nb0 + nb2 + nb4 + nb8 + nb16 + nb32 + nb64 to obtain the weights associated with each variable. This model represents a transformation of the original variables in the space of maximal difference for the group.

## Results

### Poor and Good Performers

#### Group Comparison Differences for Poor and Good Performers

**FIGURE 2:**
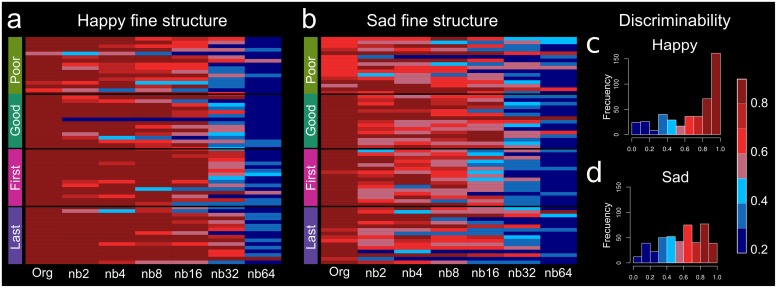
Heatmap for accuracy of response by group and uncertainty. Discrimination heatmap for accuracy concerning uncertain **(a)** happy fine structure accuracy and **(b)** sad fine structure accuracy. The darker red color represents greater discriminability of emotion and similarity with the original un-altered excerpt. On the *x*-axis, band number is represented with Org, unaltered original excerpt, to nb64. Discriminability is represented by identification accuracy with the unaltered excerpt. **(c)** Happy discriminability and **(d)** sad discriminability. The color bar represents discriminability 20th percentile bands.

Percent identification for poor (df = 6, 18, *F* = 30.76, *p* < 0.0001) and good (df = 6, 18, *F* = 79.04, *p* < 0.0001) performers was significantly different by decomposition, indicating a decrease in certainty (Figures 2a,b). Both poor and good performers used TFS for emotion identification, and discrimination performance decreased with increasing band (Supplementary Figures S1a–d). Supplementary Figures S1a, S2b show the average male poor and good identification curves, and Supplementary Figures S1c,d shows the average female poor and good identification curves. For visualization, happy and sad discriminability was represented by identification accuracy with the unaltered excerpt in 20th percentile bands (Figures S2c,d).

#### Fine Structure and Envelope Identification Differences for Happy or Sad Emotion for Poor and Good Performers

There were no apparent differences for poor performers’ TFS- or ENV-based identification of happy or sad emotion. For example, good performers did not show differences for happy TFS (df = 1, 6, *F* = 2.545, *p* = 0.1617), sad TFS (df = 1, 6, *F* = 1.494, *p* = 0.2674), happy ENV (df = 1, 6, *F* = 1.897, *p* = 0.2176), and sad ENV (df = 1, 6, *F* = 2.885, *p* = 0.1403). For good performers, happy in TFS (df = 1, 6, *F* = 7.749, *p* = 0.0318) and sad in ENV (df = 1, 6, *F* = 7.591, *p* = 0.0331) were different between male and females. Sad in TFS was significantly different between poor and good performers (df = 6, 30, *F* = 3.773, *p* = 0.0091), but happy in TFS did not reach significance (df = 6, 30, *F* = 1.912, *p* = 0.1218). Happy in ENV was significantly different between poor and good performers (df = 1, 6, *F* = 3.630, *p* = 0.0110), but sad in ENV did not reach significance (df = 1, 6, *F* = 1.881, *p* = 0.1273).

#### Discrimination Curves for Poor and Good Performers

Non-musician good and poor performers were significantly different for uncertain emotion discrimination (df = 15, 90, *F* = 1.814, *p* = 0.0445). Good and poor performers used TFS to discriminate happy and sad uncertain emotions differently, by approximately 4.01 ± 3.33% SD and 9.20 ± 6.82% SD, respectively, depending on the increasing uncertainty of stimuli (Supplementary Figures S1a–d and Discrimination curves). Males and females, irrespective of poor or good performers, used TFS to discriminate happy and sad uncertain emotions differently, by approximately 2.67 ± 1.62% SD and 2.06 ± 1.67% SD, respectively, depending on the increasing uncertainty of stimuli (Supplementary Figures S1a–d, Discrimination curves).

#### Average Discriminability A’ and Benefit of TFS and ENV for Poor and Good Performers

**FIGURE 3:**
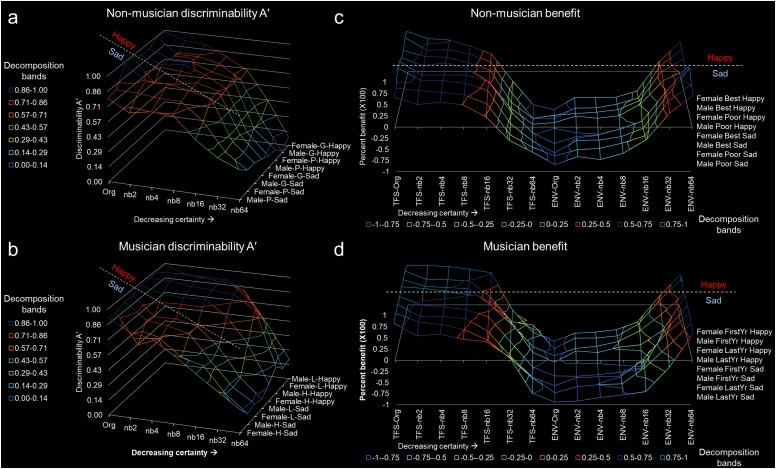
Discriminability A’ and TFS/ENV benefit to stimuli perception of uncertain emotion. **(a)** Non-musician discriminability A’ and **(b)** musician discriminability A’. **(c)** Non-musician benefit and **(d)** musician benefit. For all figures, the *x*-axis represents band number from Org to nb64. The band decompositions were associated in ranges to represent the discriminability. The *z*-axis represents sex (male or female), type of group (G – good, P – poor, L – first-year musician, H – last-year musician), and emotion type (happy or sad). Here, groups are connected to visualize the trend and pattern more readily. For example panels **(a,b)**, all groups interpret stimuli with greater certainty (i.e., greater discriminability A’ shows more blue coloring) for happy more than sad. The dashed line represents the grouping of happy and sad. Note that happy is fuller than sad, indicating high A’. For benefit panels **(c,d)**, note that for TFS there is a fuller line graph occupying more area, indicating that the majority of individuals use fine structure to discriminate uncertain emotion. Note the subtle difference between happy and sad, represented by the dashed line. Musicians benefit from TFS more than non-musicians.

The corrected non-parametric measure of discriminability (A’) was used for determining differences in emotional resolvability, and no significant difference between poor performers sad (*p* = 0.5957) and happy (*p* = 0.6612) was found. Similarly, no difference between good performers sad (*p* = 0.6712) and happy (*p* = 0.6644) was found. Figure 3a demonstrates grouped A’ for poor and good performers by uncertain emotion. Figure 3c depicts the averaged normalized benefit of TFS or ENV to the emotion discrimination curve for poor and good performers. The TFS was beneficial for emotion discriminability for poor and good performers for sad and happy stimuli. Poor performers had 0.2928 and 0.2377 benefit for happy and sad, respectively. Good performers had 0.3295 and 0.3355 benefit for happy and sad, respectively. The ENV was negatively beneficial for emotion discrimination for all stimuli for poor or good performers.

### First and Last Year Musicians

#### Group Comparison Differences for First- and Last-Year Musicians

Percent identification for first-year musicians (df = 6, 18, *F* = 71.69, *p* < 0.0001) and last-year musicians (df = 6, 18, *F* = 45.37, *p* < 0.0001) were significantly different by decomposition, indicating decrease in certainty (Figures 2a,b). Both first-year and last-year musicians using TFS for emotion identification and discrimination decreased with increasing band (Supplementary Figures S2a–f). First-year (df = 6, 18, *F* = 28.29, *p* < 0.0001) and last-year (df = 6, 18, *F* = 29.04, *p* < 0.0001) musicians resolved happy and sad emotion differently. Supplementary Figures S2a,d shows the average first- or last-year musician discrimination curves for happy and sad stimuli. Supplementary Figures S2b,c shows male and female first-year musician discrimination curves and Supplementary Figures S2e,f shows male and female last-year musician averaged discrimination curves.

#### Fine Structure and Envelope Identification Differences for Happy or Sad Emotion for First- and Last-Year Musicians

There were no apparent identification differences for first-year musicians related to TFS happy (df = 1, 6, *F* = 3.364, *p* = 0.1163), TFS sad (df = 1, 6, *F* = 0.1967, *p* = 0.6729) or ENV happy (df = 1, 6, *F* = 0.1047, *p* = 0.7573). Interestingly, first-year musicians used ENV sad significantly more for emotional resolvability (df = 1, 6, *F* = 6.058, *p* = 0.0490). Last-year musicians were significantly different for emotional resolvability for TFS happy (df = 1, 6, *F* = 14.88, *p* = 0.0084), TFS sad (df = 1, 6, *F* = 11.91, *p* = 0.0136), ENV happy (df = 1, 6, *F* = 13.66, *p* = 0.0101), and ENV sad (df = 1, 6, *F* = 14.49, *p* = 0.0089). There were significant differences in emotional resolvability between first- and last-year musicians for TFS happy (df = 6, 18, *F* = 7.585, *p* = 0.0017), TFS sad (df = 6, 18, *F* = 4.574, *p* = 0.0150), ENV happy (df = 6, 18, *F* = 4.966, *p* = 0.0110), and ENV sad (df = 6, 18, *F* = 7.816, *p* = 0.0015).

#### Discrimination Curves for First- and Last-Year Musicians

When comparing first- and last-year musicians, we found a significant difference in discriminating uncertain emotion (df = 15, 90, *F* = 4.377, *p* < 0.0001). First- and last-year musicians used TFS to discriminate happy and sad uncertain emotion differently, by approximately 2.51 ± 1.68% SD and 3.90 ± 2.30% SD, respectively, depending on the stimuli uncertainty (Supplementary Figures S1a–f, S2a–f – Discrimination curves). Males and females, irrespective of first/last year musical education used TFS to discriminate happy and sad uncertain emotion differently, by approximately 12.10 ± 4.08% SD and 4.24 ± 3.16% SD, respectively, depending on the increasing uncertainty of stimuli (Supplementary Figures S2a–f – Discrimination curves).

#### Average Discriminability A’ and Benefit of TFS and ENV for First- and Last-Year Musicians

The corrected non-parametric measure of discriminability (A’), used for determining differences in emotional resolvability, found a significant difference between first-year and last- musicians (Figure 3b). First-year musicians discriminability A’ for sad was 0.5794 and last-year musicians discriminability A’ for sad was 0.5632. However, first- and last-year musician discriminability A’ for happy was 0.7725 and 0.7805, respectively. This represents a discriminability A’ difference of 24.99% for first-year and 27.83% for last-year musicians. Figure 3b demonstrates discriminability A’ for first-year and last-year musicians by uncertain emotion. Note that the blue portion of the curve for sad is missing, indicating a lack of discriminability. Figure 3d depicts the averaged normalized benefit of TFS or ENV to the emotion discrimination curve for musicians. The TFS was beneficial for emotion discriminability for first or last year musicians for sad and happy stimuli. First year musicians had 0.4813 and 0.3013 benefit for happy and sad, respectively. Last-year musicians had 0.4652 and 0.3094 benefit for happy and sad, respectively. The ENV was negatively beneficial for emotion discrimination for all stimuli for musically educated participants.

### Poor and Good Performers Compared With First- and Last-Year Musicians

#### Group Comparison Differences and Discriminant Analysis for Non-musicians and Musicians

**FIGURE 4:**
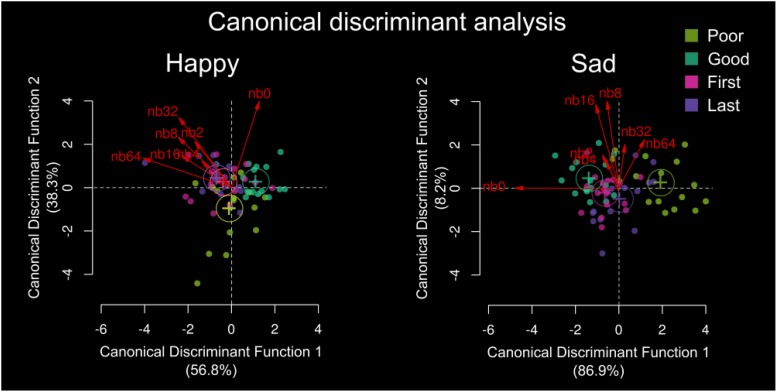
Canonical discriminant analysis for **(a)** happy and **(b)** sad uncertain emotion discrimination. Group is sorted by poor performer, good performer, first year music education and last year music education with band number from Org (nb0) to nb64 in red. The plot shows the canonical scores for the groups. Scores are indicated by points and the canonical structure coefficients are indicated by vectors from the origin. Standardized beta coefficients are given for each variable in each discriminant (canonical) function, and the larger the standardized coefficient, the greater the contribution of the respective variable to the discrimination between groups. Here, the discriminant function coefficients denote the unique contribution of each variable to the discriminant function, while the structure coefficients denote the simple correlations between the variables and the functions. For happy, the greatest standardized beta coefficients for org was Can1 = 0.6264, and for nb64 was Can1 = –0.7641. For sad, the greatest standardized beta coefficients for org were Can1 = –1.011, and for nb64 was Can1 = 0.4193.

Identification of happy differed significantly for non-musicians and musicians (df = 9, 54, *F* = 15.68, *p* < 0.0001) and by emotional resolvability (df = 6, 54, *F* = 315.5, *p* < 0.0001; Figure 4a). Identification of sad differed significantly between non-musicians and musicians (df = 9, 54, *F* = 3.526, *p* = 0.0017) and by emotional resolvability (df = 6, 54, *F* = 112.1, *p* < 0.0001; Figure 4b). Figure 4 demonstrates the separation between the different groups.

Group separation and differences were based on emotional resolvability, and Figure 4 shows the spread of separation weighted by the band decomposition as result of the canonical discriminant analysis. For happy, the greatest discriminant variables between groups were the original stimuli (Can1 = 0.6264) and nb64 (Can1 = –0.7641). For sad, the greatest discriminant variables were the original stimuli (Can1 = –1.011) and nb64 (Can1 = 0.4193). For both sad and happy, the greatest discriminant variables were the original stimuli and the hybrid stimulus nb64, indicating that the original and most uncertain stimuli were most discriminable between our groups (Figure 4).

#### Average Discriminability A’ of TFS and ENV for Non-musicians and Musicians

The differences in discriminability (A’) between non-musicians and musicians (Figures 3a,b) for happy were statistically significant (df = 1.518, 9.109, *F* = 8.796, *p* = 0.0101), as were differences in A’ as a function of emotional resolvability (df = 6, 42, *F* = 191.7, *p* < 0.0001). Differences in A’ between non-musicians and musicians (Figures 3a,b) for sad were statistically significant (df = 2.934, 17.61, *F* = 5.086, *p* = 0.0107), as were differences in A’ as a function of emotional resolvability (df = 6, 42, *F* = 156.1, *p* < 0.0001).

#### Benefit of TFS and ENV for Non-musicians and Musicians

The averaged normalized benefit of happy TFS to the emotion discrimination curve (Figures 3c,d) was statistically different between non-musicians and musicians (df = 2.383, 14.30, *F* = 6.922, *p* = 0.0060), as was the difference in benefit of happy TFS to emotional resolvability (df = 6, 42, *F* = 298.5, *p* < 0.0001). The averaged normalized benefit of sad TFS to the emotion discrimination curve (Figures 3c,d) was not statistically different between non-musicians and musicians (df = 2.643, 15.86, *F* = 1.826, *p* = 0.1870), although the contribution to emotional resolvability was statistically significant (df = 6, 42, *F* = 333.0, *p* < 0.0001). The averaged normalized benefit of happy ENV to the emotion discrimination curve (Figures 3c,d) was not statistically different between non-musicians and musicians (df = 2.638, 15.83, *F* = 1.708, *p* = 0.2087), although the contribution to emotional resolvability was statistically significant (df = 6, 42, *F* = 342.1, *p* < 0.0001). The averaged normalized benefit of sad ENV to the emotion discrimination curve (Figures 3c,d) was statistically different between non-musicians and musicians (df = 2.909, 17.45, *F* = 10.05, *p* = 0.0005), as was the difference in benefit of sad ENV to emotional resolvability (df = 6, 42, *F* = 450.0, *p* < 0.0001).

## Discussion

The objective of the present experiment was to investigate certain and uncertain emotion in musical sounds, and determine if non-musicians and musicians resolve emotion differently. Here, stimuli that varied in emotional certainty were presented in a happy/sad interval forced-choice discrimination psychophysical task. There are three results of considerable interest: *First*, TFS information was essential to discriminating emotion in sound. *Second*, different emotional resolvability curves were found to depend on whether participants were poor or good performers and on year of musical education. *Third*, non-musicians used less TFS and had reduced emotional resolvability curves compared to musicians. The aim of the present experiment was to understand the cues used to resolve emotional signals at threshold and how they differ between non-musicians and musicians.

### Resolving Emotion Using Psychoacoustic Cues

Emotion in sound is transmitted through TFS and ENV modulations. In a groundbreaking study, Lieberman and Michaels (1962) found that pitch aided in the identification of emotional content by 44% while amplitude cues added only 3% more. The present study found TFS cues essential and beneficial to discriminating emotion in musical excerpts, whether individuals were good performers, poor performers, or had musical training. The results indicate that TFS cues are required for resolving emotion in sound and individuals differ in their perceptive ability to discriminate these cues. Furthermore, happy emotion was discriminated with higher accuracy than sad emotion for all groups (Figures 2a, 3 and Supplementary Figures S1, S2). This is most likely due to individuals using major mode and the fast tempo of tones for discriminating emotion in sound, which are prominent in happy stimuli (Peretz et al., 2001; Hopyan et al., 2016). However, how these cues determine specific emotions and are perceived by individuals is not completely understood. For example, individuals differ in their tendency to report the co-occurrence of discrete emotions of the same valence (Barrett, 1998). Individuals vary in the extent to which they distinguish between like-valence discrete emotions or did not distinguish between like-valence emotions when reporting on their subjective experience (Barrett, 1998). The results indicate that individuals are reporting several affective states together, or it may indicate they are not distinguishing between distinct emotional states. The aforementioned manuscript (Barrett, 1998) bolstered support for both the theory of discrete emotion, where individuals label emotions based on determining a subjective level of arousal, and the dimensional theory of emotion, where individuals focus on the subjective emotional experience dimensionalized by valence, arousal, and intensity of the affective state (Russell, 1980; Barrett, 1998). The present study found emotional resolvability changed as a function of altering the TFS content of the musical excerpt, revealing that an essential cue to discriminating emotion is fine structure. Recently, a study investigating the similarities/dissimilarities of emotion in music and speech prosody found that the psychoacoustic features implicated were loudness, tempo and speech rate, melodic and prosodic contour, spectral centroid, and sharpness (Coutinho and Dibben, 2013). In contrast, the features distinct to music and speech were spectral flux and roughness, respectively. Here, the authors indicated that emotional cues in sound are encoded as psychoacoustic spatiotemporal patterns, which for music and speech rely heavily on their “shared acoustic profiles” (Coutinho and Dibben, 2013). We encourage research into determining what constitutes an emotion from non-emotion sound (Pfeifer, 1988; Barrett, 1998; LeDoux, 2000; Phan et al., 2002; Wildgruber et al., 2005; Koelsch, 2014), which will enable a more thorough classification of the neurobiology of emotion. Future studies should further explore the psychoacoustic foundations of emotion.

### Musicians Compared With Non-musicians

Musicians discriminate emotion differently, likely due to their unique education. For example, in the speech-in-noise and hearing-in-noise test, musicians perform significantly better than non-musicians, derived in part from musicians’ enhanced working memory and frequency discrimination (Parbery-Clark et al., 2009a, b). Musicians in the present study discriminated emotion in sound differently than non-musicians by using more TFS through each of the nb decompositions. Within the group of musicians, last-year musically educated individuals discriminated happy or sad excerpts somewhat differently than first-year musically educated individuals. Although the greatest difference was in male and female musicians, the difference between musicians and non-musicians reveals the most, as musicians benefited more from TFS components. For example, musicians discriminating happy or sad excerpts utilized more TFS irrespective of whether individuals were in the first or last year of their music education. Recent discrimination tasks bolster these results. In a study where participants were tasked to detect frequency changes in quiet and noisy conditions, the acoustic change complex, a type of late auditory evoked potential, showed a larger P2’ amplitude in musicians than in non-musicians (Liang et al., 2016). Moreover, in a task where target speech and competing speech were presented with either their natural F0 contours or on a monotone F0, and with F0 difference between the target and masker systematically varied, F0 discrimination was significantly better for musicians (Madsen et al., 2017). Most of these frequency discrimination tasks indicate that musicians have an enhanced ability to perceive or discriminate TFS or fine structure components. Future studies should expand the range and variety of emotion discrimination paradigms, to explore differences between musicians and non-musicians.

### Study Limitations and Future Directions

The present study concentrated on analyzing two emotions elicited by classical music excerpts. Constraining the variety of emotion to a dichotomous task is artificial, but aides in discerning how the basic components of sound cue emotion. Future studies should analyze the diverse emotional repertoire that exists in humans. The present study analyzed non-musicians and individuals with musical education. We chose these groups based on previous literature (Micheyl et al., 2006; Musacchia et al., 2007; Wong et al., 2007; Parbery-Clark et al., 2009a, b; Kraus and Chandrasekaran, 2010; Strait et al., 2010; Bas̨kent and Gaudrain, 2016; Liang et al., 2016; Mandikal Vasuki et al., 2016; Coffey et al., 2017; Madsen et al., 2017), suggesting music training would influence acoustic perception in the emotional resolvability task. This manuscript found that years in music education significantly affected emotional resolvability (*F*_15__,__90_ = 4.377, *p* < 0.0001), with advanced musicians using more fine structure to discriminate happy uncertain emotion by 2.51 ± 1.68%. Future studies could analyze different musicians (piano versus string) to determine if emotional resolvability differences are associated with the type of instrument training. The present manuscript assessed males and females separately, as it is known that gender differences in the perception of non-target emotions (incorrect) are greater for men than women (Fischer et al., 2018). Further, our entire cohort of subjects (*n* = 64) was derived from a Spanish speaking population. In this regard, identifying emotion is easier for listening individuals with similar cultural and language backgrounds (Waaramaa and Leisiö, 2013) and a second language is known to interfere with emotion recognition from speech prosody (Bhatara et al., 2016). The repertoire of music used in the present study was classical, which was familiar to all participants; therefore, we believe the effect due to cultural background should be minimal. Future studies could further assess these confounding variables to determine their affect on uncertain musical emotion.

## Data Availability

Data and sound files used in this work can be downloaded in an anonymized format from the Open Science Framework: Manno, Francis A. M. 2018. “Music Psychophysics.” OSF. November 20. https://osf.io/8ws7a.

## Ethics Statement

The studies involving human participants were reviewed and approved by the Internal Review Board of the Instituto de Neurobiología, Universidad Nacional Autónoma de México. The participants provided their written informed consent to participate in this study.

## Author Contributions

FM designed the experiments, interviewed participants, executed experiments, analyzed the data, and wrote the manuscript. FM and RC assisted in experimental design and analysis, and revised the document. CL and FB supervised the experiments, assisted with data curation and analysis, and assisted in writing the manuscript.

## Conflict of Interest Statement

The authors declare that the research was conducted in the absence of any commercial or financial relationships that could be construed as a potential conflict of interest.

## Footnotes

1 https://github.com/rcruces/2019_uncertain_emotion_discrimination

2 www.brams.umontreal.ca/peretz

3 http://psychtoolbox.org

## Supplementary Material

FIGURE S1 Discrimination curves and discrimination normalized ratio indices for non-musicians. Discrimination curves of percent identification of happy or sad stimuli. **(a)** Average male poor performers, **(b)** average male good performers, **(c)** average female poor performers, and **(d)** average female good performers. Sad curve represented with blue line and happy curve represented with red line. The discrimination normalized ratio of stimuli identification of happy or sad stimuli. **(e)** Male poor performers, **(f)** male good performers, **(g)** female poor performers, and **(h)** female good performers. Sad discrimination ratios are represented in red and happy discrimination ratios are represented in blue.

FIGURE S2 Discrimination curves and discrimination normalized ratio indices for musicians in their first or last year of study. Discrimination curves of percent identification of happy or sad stimuli. **(a)** Average first year (low) and **(d)** average last year music education (high). Male **(b)** and female **(c)** first year and male **(e)** and female **(f)** last year music education. The discrimination normalized ratio of stimuli identification of happy or sad stimuli **(g)** through **(j)**. Sad curve represented with blue line and happy curve represented with red line. **(g)** Male first year and **(h)** male last year. **(i)** Female first year and **(j)** female last year.

TABLE S1 Montreal emotional identification task acoustic stimuli.

DATA SHEET S1 Contains extended analyses.

DATA SHEET S2 Contains the original data used for all analyses in a csv file.
