# Supplementary figures and images for: Uncertain Emotion Discrimination Differences Between Musicians and Non-musicians Is Determined by Fine Structure Association: Hilbert Transform Psychophysics
Source: Front Neurosci. 2019 Sep 18;13:902. doi: 10.3389/fnins.2019.00902 (PMC6759500; doi:10.3389/fnins.2019.00902)

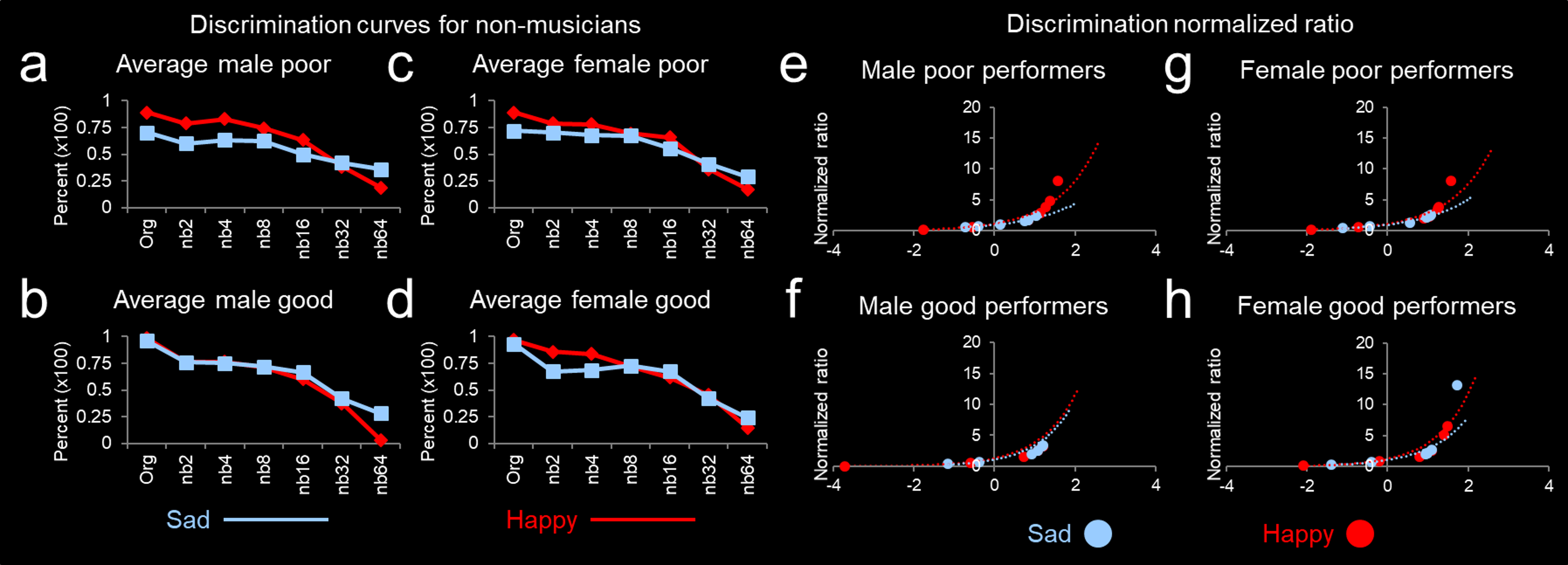

Supplement: FIGURE S1 — Discrimination curves and discrimination normalized ratio indices for non-musicians. Discrimination curves of percent identification of happy or sad stimuli. (a) Average male poor performers, (b) average male good performers, (c) average female poor performers, and (d) average female good performers. Sad curve represented with blue line and happy curve represented with red line. The discrimination normalized ratio of stimuli identification of happy or sad stimuli. (e) Male poor performers, (f) male good performers, (g) female poor performers, and (h) female good performers. Sad discrimination ratios are represented in red and happy discrimination ratios are represented in blue. [file Image_1.tiff]

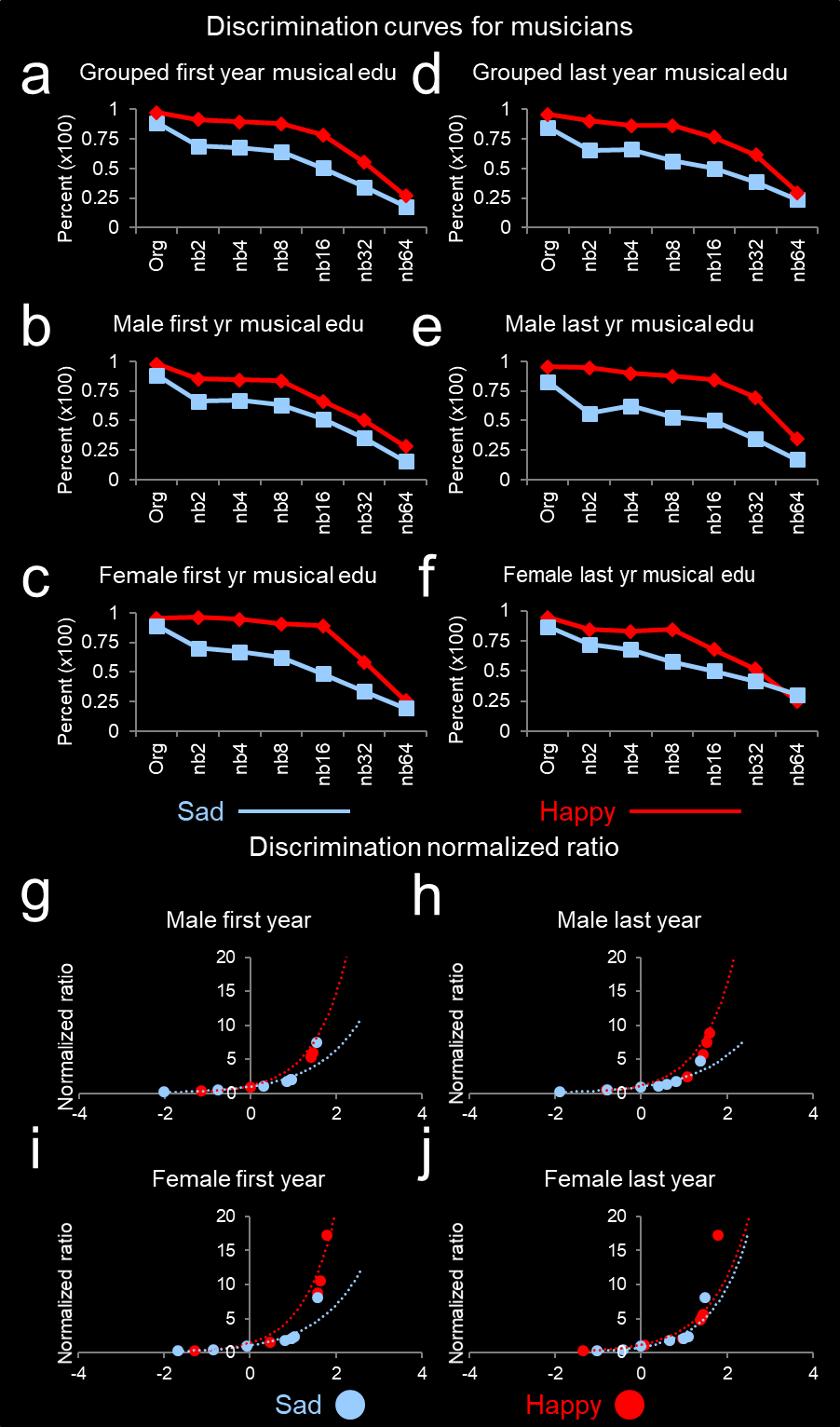

Supplement: FIGURE S2 — Discrimination curves and discrimination normalized ratio indices for musicians in their first or last year of study. Discrimination curves of percent identification of happy or sad stimuli. (a) Average first year (low) and (d) average last year music education (high). Male (b) and female (c) first year and male (e) and female (f) last year music education. The discrimination normalized ratio of stimuli identification of happy or sad stimuli (g) through (j). Sad curve represented with blue line and happy curve represented with red line. (g) Male first year and (h) male last year. (i) Female first year and (j) female last year. [file Image_2.tiff]
