# Supplementary material for: Uncertain Emotion Discrimination Differences Between Musicians and Non-musicians Is Determined by Fine Structure Association: Hilbert Transform Psychophysics
Source: Front Neurosci. 2019 Sep 18;13:902. doi: 10.3389/fnins.2019.00902 (PMC6759500; doi:10.3389/fnins.2019.00902)
Supplement: TABLE S1 — Montreal emotional identification task acoustic stimuli. [file Data_Sheet_1.pdf]

Uncertain emotion discrimination differences between musicians and nonmusicians is determined by fine structure association: Hilbert transform psychophysics

Updated on 15 August, 2019

Authors: Francis A.M. Manno, Raul R. Cruces, Condon Lau, Fernando A. Barrios
Contact: francis.manno@sydney.edu.au, raulrcruces@inb.unam.mx, fbarrios@unam.mx, condon.lau@cityu.edu.hk

OSF: https://osf.io/8ws7a
Keywords: emotion; psychophysics; modulation; fine structure; envelope; frequency; amplitude

Abstract

Humans perceive musical sound as a complex phenomenon, which is known to induce an emotional response. The cues used to perceive emotion in music have not been unequivocally elucidated. Here, we sought to identify the attributes of sound that confer an emotion to music and determine if professional musicians have different musical emotion perception than non-musicians. The objective was to determine which sound cues are used to resolve emotional signals. Happy or sad classical music excerpts modified in fine structure or envelope conveying different degrees of emotional certainty were presented. Certainty was determined by identification of the emotional characteristic presented during a forced-choice discrimination task. Participants were categorized as good or poor performers (n = 32, age 21.16 ± 2.59 SD) and in a separate group as musicians in the first or last year of music education at a conservatory (n = 32, age 21.97 ± 2.42). We found that temporal fine structure information is essential for correct emotional identification. Non-musicians used less fine structure information to discriminate emotion in music compared with musicians. The present psychophysical experiments revealed what cues are used to resolve emotional signals and how they differ between non-musicians and musically educated individuals.

Code and Methods

The code for **figure 2** and **figure 4** can be found in `Final_figures.R`.

R 3.4.4, required packages

```r
library(gplots)
library(gridExtra)
library(MASS)
library(pander)
library(magrittr)
library(dplyr)
library(ggplot2)
library(RCurl)
library(scatterplot3d)
library(klaR)
library(pander)
library(candisc)
```

Group Heatmaps

This was created calculating the accuracy of each subject per stimuli. First, stimuli where binarized based in the answer of **Fine structure emotion**, with 1 if is correct and 0 when wrong. The total was divided by the total amount of stimuli or each category (nb0...nb64).

Accuracy for both emotions: All subjects

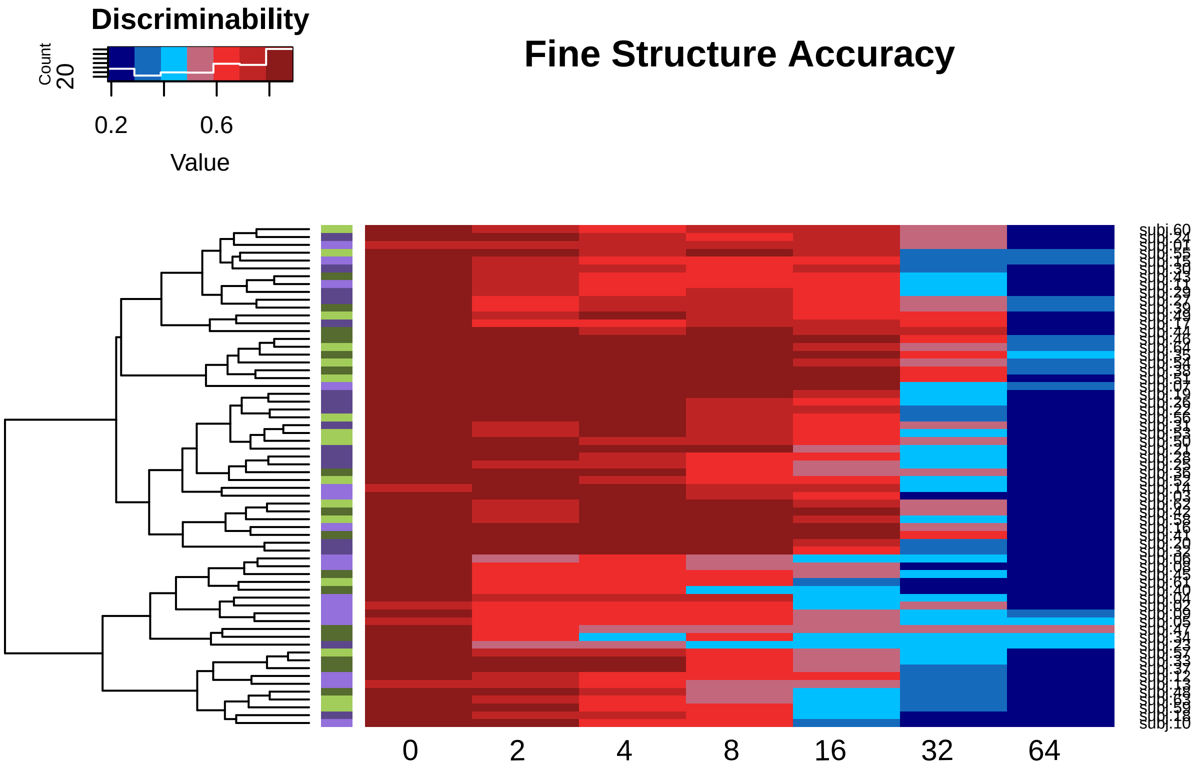

Table 1.

Table 1: Table continues below

	N	age.mean	age.sd	years.mean	years.sd	range.1
**M.good**	8	20.16	2.11	0	0	18.2
**F.good**	8	22.64	3.58	0	0	18.7
**M.poor**	8	21.11	2.27	0	0	18.8
**F.poor**	8	20.74	2.11	0	0	18.6
**M.first**	8	20.89	1.74	2	0	18.9
**F.first**	8	20.56	1.17	2	0	18.8
**M.last**	8	23.16	2.01	8.38	2.26	19.7
**F.last**	8	23.27	3.34	7.12	2.1	20

	range.2	correct.mean	correct.sd	correct.N
**M.good**	25	97.66	2.21	31.25
**F.good**	29.2	95.31	2.36	30.5
**M.poor**	25.8	80.08	2.86	25.62
**F.poor**	24.8	80.86	4.24	25.88
**M.first**	24	93.36	2	29.88
**F.first**	22.8	92.58	5.52	29.62
**M.last**	26.1	89.45	3.31	28.62
**F.last**	29.8	91.02	4.24	29.12

Fine Structure Accuracy by Gender

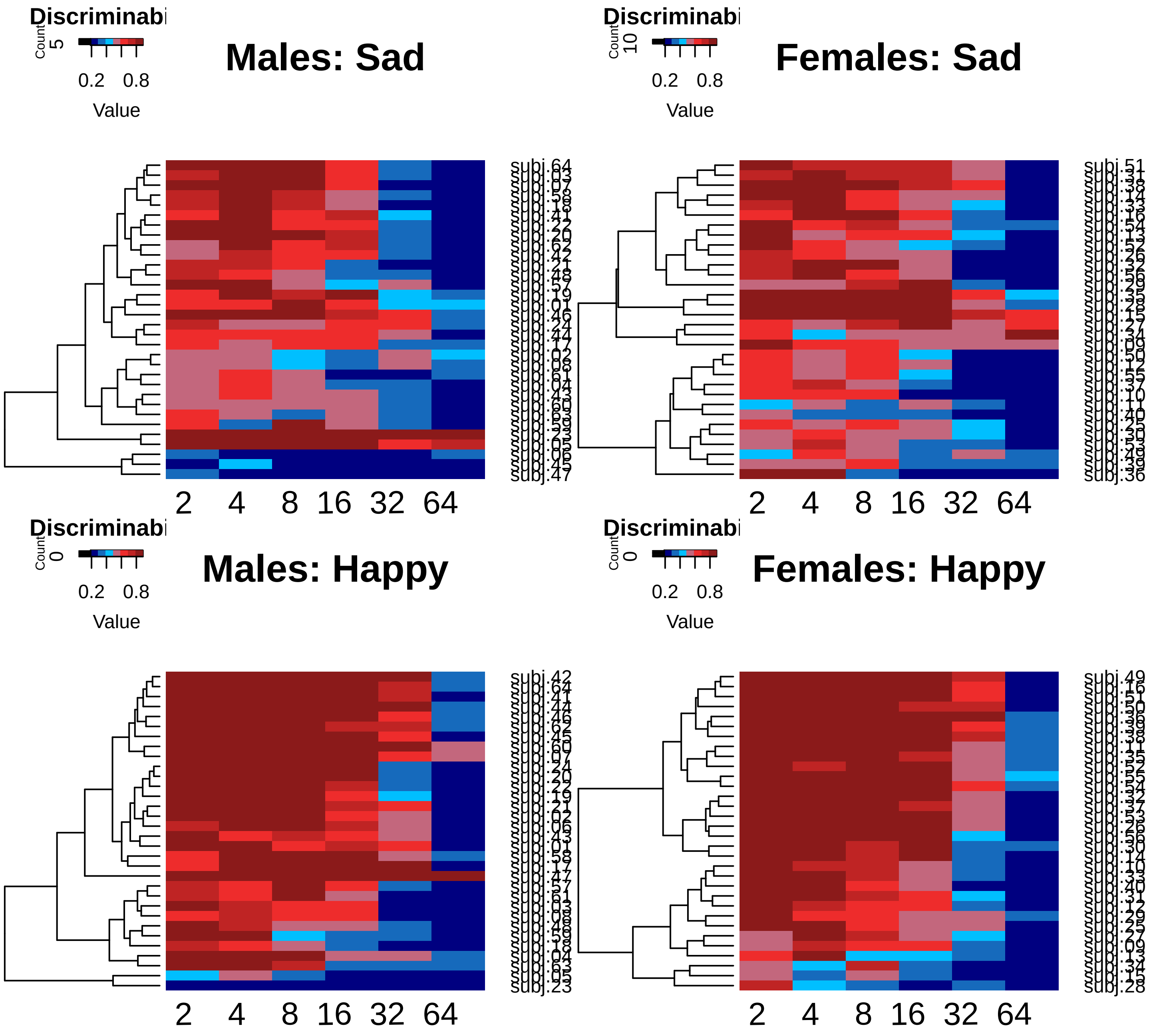

Figure 2. Accuracy and discriminability concerning Happy and Sad

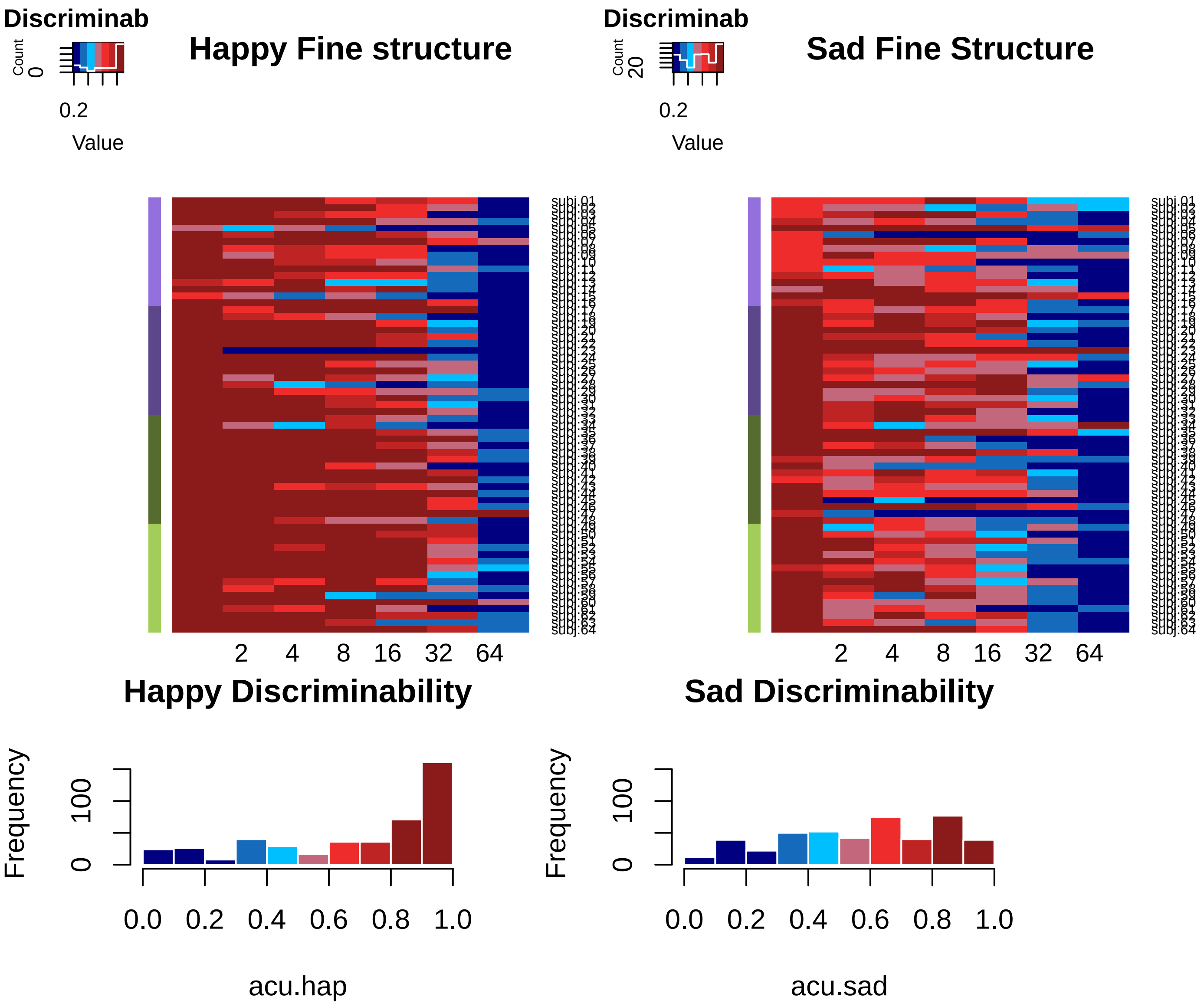

Accuracy calculaded by subjects and group

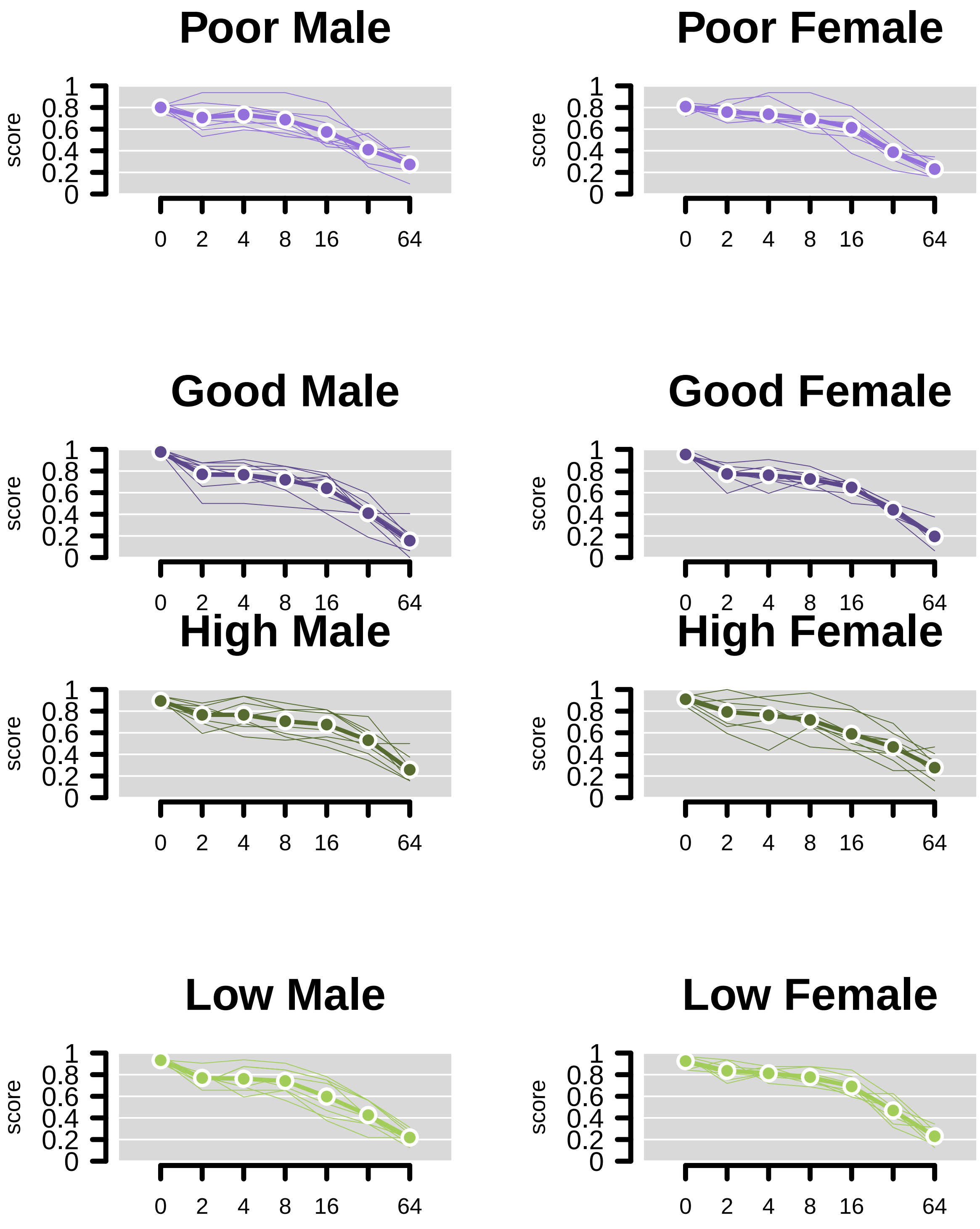

ANOVA: Comparisons between groups

Is accuracy in the response given by the class belonging and gender?

Table 3: ANOVA, accuracy by class

	Df	Sum Sq	Mean Sq	F value	Pr(>F)
**factor(cases$class)**	3	0.2268	0.0756	60.55	5.292e-18
**factor(cases$gender)**	1	6.104e-05	6.104e-05	0.04888	0.8258
**Residuals**	59	0.07367	0.001249	NA	NA

- **factor(cases$class)**:

	diff	lwr	upr	p adj
**high-best**	-0.0625	-0.09553	-0.02947	3.147e-05
**low-best**	-0.03516	-0.06819	-0.002127	0.03271
**poor-best**	-0.1602	-0.1932	-0.1271	1.795e-11
**low-high**	0.02734	-0.005686	0.06037	0.1383
**poor-high**	-0.09766	-0.1307	-0.06463	6.858e-10
**poor-low**	-0.125	-0.158	-0.09197	1.813e-11

- **factor(cases$gender)**:

	diff	lwr	upr	p adj
**M-F**	0.001953	-0.01572	0.01963	0.8258

Linear Discriminant Analysis: Happy

$Group b0 + nb2 + nb4 + nb8 + nb16 + nb32 + nb64$

best	high	low	poor
0.2031	0.1406	0.07812	0.125

Table 7: LDA happy: Observed vs. Predicted Frequencies

	best	high	low	poor	Sum
**Predicted best**	13	2	4	2	21
**Predicted high**	1	9	5	2	17
**Predicted low**	1	4	5	4	14
**Predicted poor**	1	1	2	8	12
**Sum**	16	16	16	16	64

Happy total % correct: 0.546875

Table 8: LDA sad: Observed vs. Predicted Proportions %

	best	high	low	poor	Sum
**Predicted best**	0.2031	0.03125	0.0625	0.03125	0.3281
**Predicted high**	0.01562	0.1406	0.07812	0.03125	0.2656
**Predicted low**	0.01562	0.0625	0.07812	0.0625	0.2188
**Predicted poor**	0.01562	0.01562	0.03125	0.125	0.1875
**Sum**	0.25	0.25	0.25	0.25	1

Table 9: Happy: group means by LDA

type	LDA1	LDA2	LDA3
best	-1.126	-0.2657	0.04361
high	0.6722	-0.4348	0.2395
low	0.3626	-0.262	-0.317
poor	0.09148	0.9625	0.03394

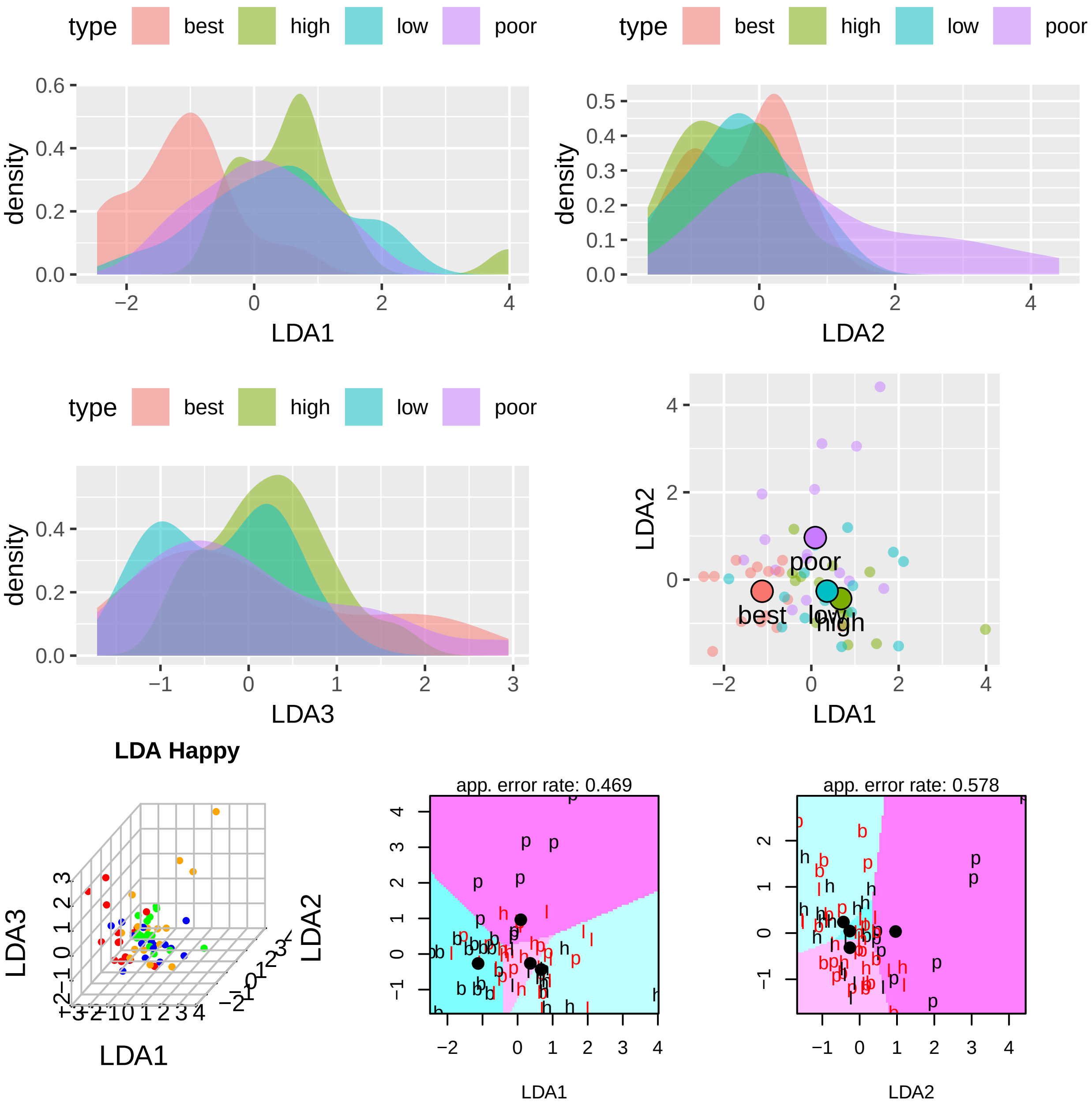
type
best
high
low
poor
density
LDA1
LDA2
LDA3
best
low
high
poor
LDA Happy
app. error rate: 0.469
app. error rate: 0.578

Linear Discriminant Analysis: Sad

best	high	low	poor
0.1562	0.125	0.1562	0.2031

Table 11: LDA sad: Observed vs. Predicted Frequencies

	best	high	low	poor	Sum
**Predicted best**	10	0	1	2	13
**Predicted high**	2	8	4	0	14
**Predicted low**	4	4	10	1	19
**Predicted poor**	0	4	1	13	18
**Sum**	16	16	16	16	64

Sad total % correct: 0.640625

Table 12: LDA sad: Observed vs. Predicted Proportions %

	best	high	low	poor	Sum
**Predicted best**	0.1562	0	0.01562	0.03125	0.2031
**Predicted high**	0.03125	0.125	0.0625	0	0.2188
**Predicted low**	0.0625	0.0625	0.1562	0.01562	0.2969
**Predicted poor**	0	0.0625	0.01562	0.2031	0.2812
**Sum**	0.25	0.25	0.25	0.25	1

Table 13: Happy: group means by LDA

type	LDA1	LDA2	LDA3
best	-1.362	0.4542	0.1613
high	0.04957	-0.4957	0.3254
low	-0.6249	-0.2197	-0.4475
poor	1.937	0.2612	-0.03927

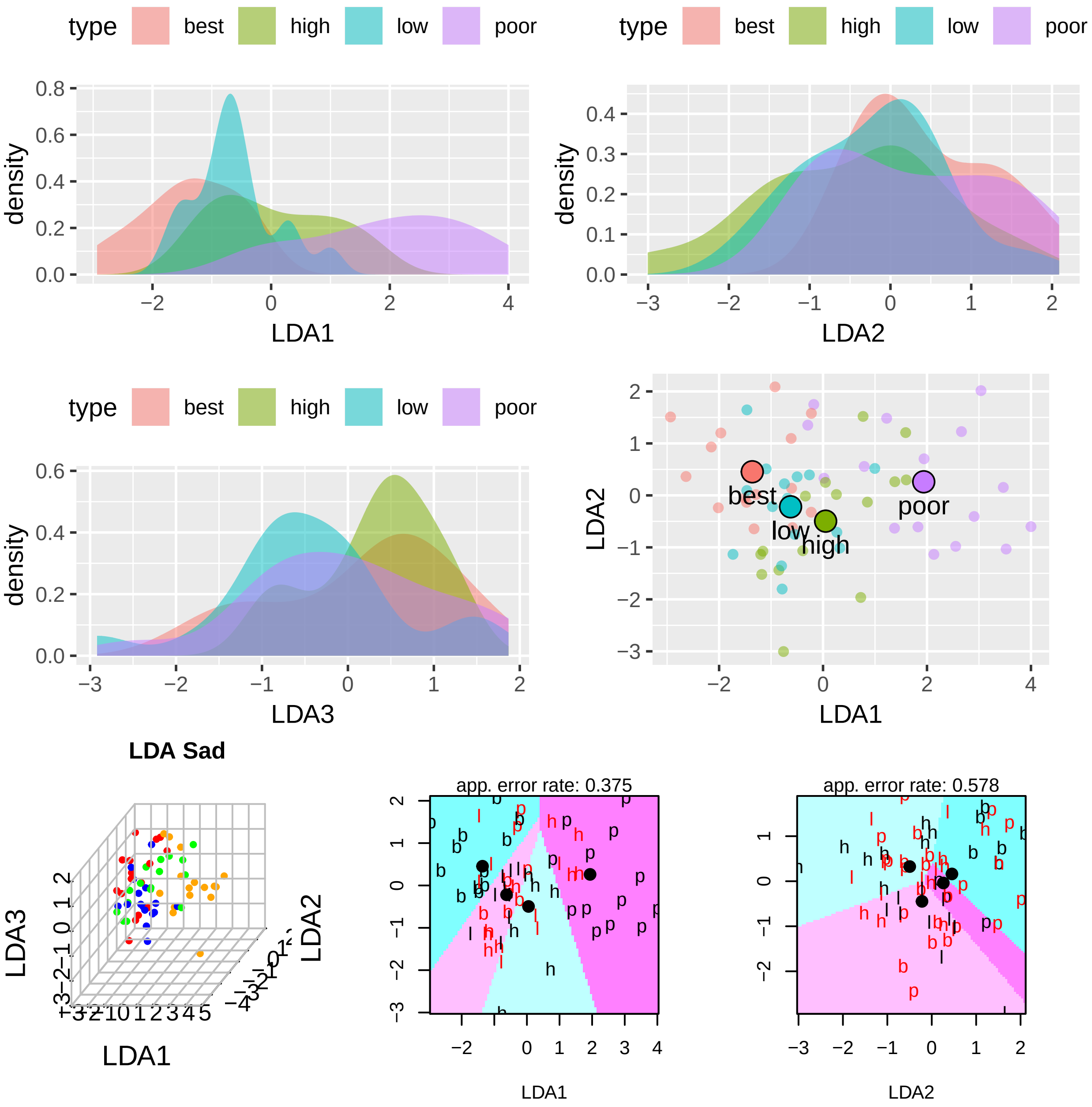
type
best
high
low
poor
density
LDA1
LDA2
LDA3
best
low
high
poor
LDA Sad
LDA3
LDA1
LDA2
app. error rate: 0.375
app. error rate: 0.578
LDA1
LDA2

Figure 4. Cannonical Discriminant Analysis (CDA)

Two generalized canonical discriminant analysis was compute using the multivariate linear model:

$$Group\ nb0 + nb2 + nb4 + nb8 + nb16 + nb32 + nb64$$

to obtain the canonical scores and vectors, one for HAPPY and the other for SAD. It represents a transformation of the original variables in the scpace of maximal differences for the group. THe biplot shows the canonical scores for the groups defined by the term as points and the canonical structure coefficients as vectors from the origin.

Standardized beta coefficients are given for each variable in each discriminant (canonical) function, and the larger the standardized coefficient, the greater is the contribution of the respective variable to the discrimination between groups. However, these coefficients do not tell us between which of the groups the respective functions discriminate.

Happy standardized coefficients

Table 14: Happy CDA standardized coefficients

	Can1	Can2	Can3
**nb0**	0.6264	0.7113	-0.3907
**nb2**	-0.1806	0.3608	0.1146
**nb4**	0.507	-0.6772	-0.4067
**nb8**	-0.5025	0.3103	-0.6578
**nb16**	-0.2266	-0.4923	-0.2479
**nb32**	-0.05239	0.7701	1.224
**nb64**	-0.7641	-0.08169	-0.24

Sad standardized coefficients

Table 15: Sad CDA standardized coefficients

	Can1	Can2	Can3
**nb0**	-1.011	-0.05111	0.04974
**nb2**	-0.01097	-0.1844	0.3217
**nb4**	0.2952	-0.4591	-0.4602
**nb8**	-0.08152	0.8909	1.472
**nb16**	-0.2719	0.5762	-1.304
**nb32**	0.06616	-0.4042	-0.1513
**nb64**	0.4193	0.4013	-0.2076

The discriminant function coefficients denote the unique contribution of each variable to the discriminant function, while the structure coefficients denote the simple correlations between the variables and the functions

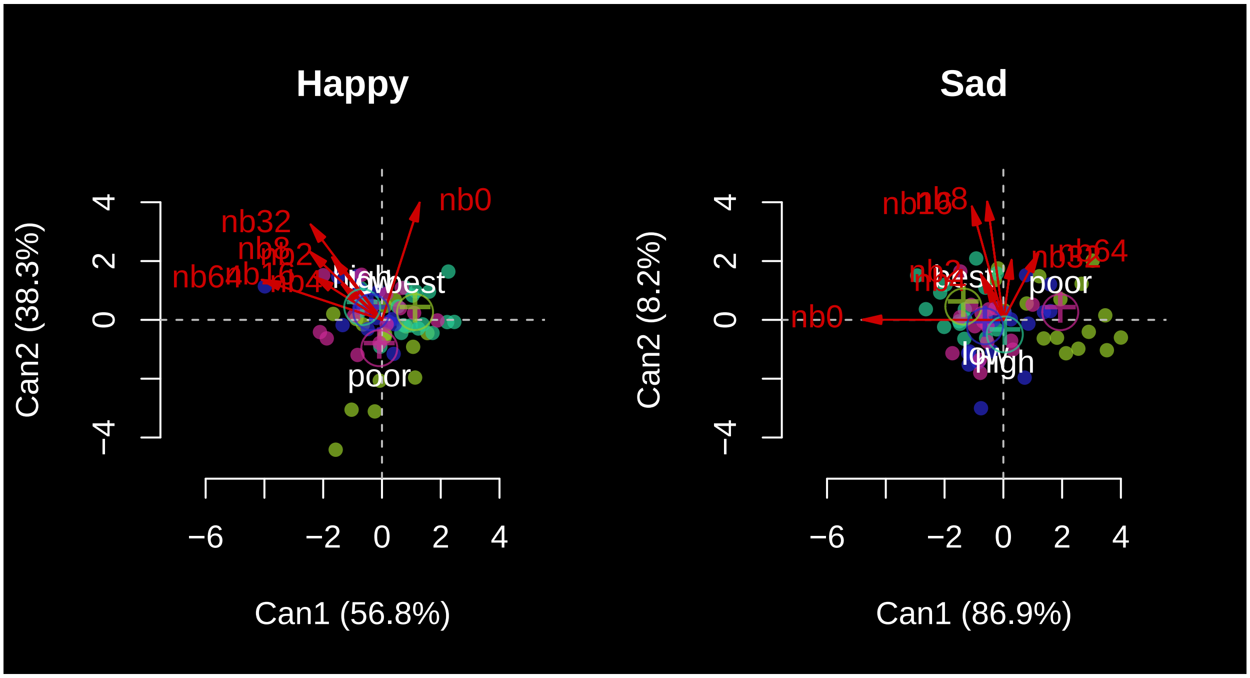
Happy
Can2 (38.3%)
Can1 (56.8%)
nb0
nb32
poor
Sad
Can2 (8.2%)
Can1 (86.9%)
nb0
poor
low
high
best
